# Editorial: The Grammar-Body Interface in Social Interaction

**DOI:** 10.3389/fpsyg.2022.875696

**Published:** 2022-04-01

**Authors:** Simona Pekarek Doehler, Leelo Keevallik, Xiaoting Li

**Affiliations:** ^1^Institute of Language Sciences, Université de Neuchâtel, Neuchâtel, Switzerland; ^2^Department of Culture and Society, Linköping University, Linköping, Sweden; ^3^Department of East Asian Studies, University of Alberta, Edmonton, AB, Canada

**Keywords:** conversation analysis, multimodality, grammar-in-interaction, interactional linguistics, gesture, gaze, posture

Human communication rests on a complex ecology of multiple resources that are orchestrated for collaborative meaning-making and coordination of social action. The aim of this Research Topic is to analyze how grammar and the body interface in naturally occurring interaction. The contributions draw on conversation analysis and interactional linguistics to demonstrate how verbal and bodily conduct is intricately intertwined: they mutually elaborate each other and are variably synchronized to achieve communicative goals. A distinctive feature of the studies is that they offer collection-based analyses of a range of grammar-body assemblies: *recurrent* simultaneous or successive combinations of grammatical constructions and bodily behavior. Taken together, they offer a rich demonstration of how analyzing language use in its full local ecology has the potential of deepening, if not revising, our very understanding of language. In this editorial, we will organize the studies into four sections as described below.

## Multimodal Action Formats

Several studies take as their starting point a specific linguistic structure and show how it is systematically coupled with precise multimodal conduct, in particular the deployment of gestures and gaze. These studies demonstrate the routinized character of language-body assemblies that accomplish specific actions in interaction (the authors are listed in alphabetical order).

Studying conversational data from Czech, French, Hebrew, Mandarin, and Romanian, Pekarek Doehler et al. identify a recurrent multimodal assembly through which speakers preface a dispreferred response to various types of sequence-initial actions: The practice involves a turn-initial expression corresponding to English “I don't know”/“dunno” coupled with gaze aversion from the prior speaker. By evidencing how grammar and body interface in related ways across a diverse set of languages, the findings open a window into cross-linguistic, cross-modal, and cross-cultural consistencies in human interactional conduct.

Focusing on the use of gesture in turn-continuing practices with the connective å* sen* “and then” in Swedish multi-party conversations, Rönnqvist and Lindström identify a recurrent multimodal trajectory: syntactic completion of a first unit + retracted gesture; link to prior talk and upcoming talk with “and then” followed by the core content of the continuation + a redeployed gesture; and finally, syntactic completion of the continuing unit + retracted gesture to a rest position. They thereby add to our understanding of multimodal practices involved in turn-continuation.

In her paper on Finnish, Stevanovic scrutinizes sequences of decision-making, focusing on positive assessments formulated with the particle *ihan* “quite” in response to proposals. She demonstrates that qualitatively different and variably synchronized embodied behavior, or lack of it, designs the action as either an in-principle acceptance, conceding, or making a joint decision. This analysis highlights the role of body movements in action formation.

The paper by Stoenica and Fiedler shows that one of the most frequent phrases in French talk-in-interaction, the turn-final *tu vois* ‘you see’, is systematically coupled with the speaker's gaze directed to the recipient, thus constituting a multimodal practice to elicit a sequentially relevant response. The study points out how various modalities are coordinated at turn completion.

Drawing on data from self-defense training in German, Stukenbrock analyzes recurrent couplings of demonstratives and gestures, which she argues are socially sedimented multimodal gestalts that may be subject to transformations in the course of multiple repetitions. In the case under scrutiny, repetition leads to the emergence of a new, reduced multimodal format, which is locally routinized but neither grammatical nor grammaticalized. The study enhances our knowledge on the development of multimodal assemblies over time.

Zinken et al. investigate the uses of impersonal deontic statements such as “It is not allowed to do this” when a rule is breached in board games. These impersonal deontic statements accomplish the action of instruction in that they serve as an account for having enforced a game rule and impart knowledge to less knowledgeable players for the future.

## Contextually Sensitive Syntax in Embodied Interaction

A further set of studies consider the intertwinement of syntactic structures with bodily matters, such as tasting, touching, or being under physical strain, and the related materialities. Among other things, they show that various “suspended” or “truncated” formats allow participants to accomplish locally specific actions. The main argument is that syntactic patterns emerge as contextually sensitive structures that are very different from those conjured up as “full” sentences.

Analyzing guided tours in French, Italian, and German, De Stefani demonstrates how – often self-standing – *if-*clauses are used by tour guides to organize visitors' attention focus on a given object of interest. Multimodal analysis documents the embodied-action projection capacity of such clauses, as they invite co-participants to physically orient to a material object present in the environment. The study reveals how the *if*-clause is adjusted in the very course of its production to co-participants' physical (re)positioning in space.

Hofstetter et al. discuss the relationship between talking in English or Swedish and bodily strain, analyzing the practice of temporarily suspending syntax while the speaker is accomplishing a physically challenging task. They argue that this is a resource available across contexts to render prominence to the strained body but also maintain rights to resume talk. The study begins to explore how the speaker's body, hitherto relatively ignored in studies of syntax, is implicated in the production of clauses.

Deppermann and Gubina focus on the “seemingly paradoxical package” of the “lean syntax” darf/kann ich? (“may/can I?”)-format indexing low agency and the concurrent embodied actions exerting high agency in German interaction. They report two ways in which the grammatical format and accompanying embodied actions are coordinated, and variably contribute to the treatment of the activity as either probable or certain. Their findings demonstrate the interrelatedness of grammar, bodily actions, and sequential position, as well as the significance of embodied agency.

Mondada's paper examines the intertwinement of embodied practices and emerging Italian syntax, permeated by the sensing body at a tasting session in which the participants are engaged in talking about sensorial features while experiencing them. The study shows how perceptive actions are embedded in the ongoing talk, and how they may affect its smooth progressivity. The choice of syntactic formats is related to the complex ecology of embodied actions, namely to publicly accountable ways of sensing material objects, to ways of addressing the audience, and to visible references to documents that normatively define tasting descriptors.

Skogmyr Marian examines the use of verbally incomplete utterances in complaints about third parties or various situations in French interaction. The findings show that in the initiation of complaints, the speaker leaves utterances verbally incomplete and displays negative stance through bodily-visual conduct; at the end of complaint reports, verbally incomplete utterances are deployed as a summary assessment of the complaint.

## Gesture and Local Meaning-Making in Interaction

Scrutinizing the use of gestural resources in interaction, the authors of the following studies demonstrate the semiotic relevance of specific gestures alongside lexicon and grammar in situated meaning-making. These gesture-language assemblies impact such central aspects as expressing modality, representation of meaning, and stance-taking.

Eskildsen's article examines an embodied object-transfer construction produced by a novice L2 speaker in an English-as-a-Second-Language classroom. The embodied object-transfer construction consists of linguistic structures (e.g., “he told me the story”) and “object-transfer gestures” (consisting of pointing gestures and gestures indicating movement). The L2 speaker's flexible (re-)uses of object-transfer gestures demonstrate the embodied nature of L2 interactional competence.

In her paper on hairdresser-client interactions in French, Horlacher discusses how similarly formatted negative utterances function as either instructions or directives, depending on where in the hairdresser service they are deployed and whether the client is touching their hair or not. Only the latter elicit immediate hairdresser action in response, thus functioning as directives. This highlights the link between materialities, touch, grammar, and action.

Marrese et al. explore the role of the palm-up (PU) gesture in argument sequences, and particularly when participants reach an “impasse” with opposing stances. In this sequential environment, participants produce the PU gesture to pursue a previously established position, and to index the obviousness of that position. The function of the PU gesture is linked to specific grammatical features in American English. This regularity points toward an embodied conceptualization of grammar related to epistemicity.

Urbanik and Svennevig investigate how physical actions are represented through both verbal structures in Norwegian and action-depicting gestures in construction site interaction. Participants use both generic depictions which represent actions as general types, and contextualized depictions which include deictic references or iconic representations. The two types of depictions accomplish different interactional goals, such as pre-empting understanding problems and facilitating understanding of action specifics. The study underlines the relevance of temporal organization of gestures in relation to talk.

## The Body in a Linguistic Ecology

The last set of papers empirically evidences how intricately language and the body interface in situated face-to-face interaction. Two of these papers start from a concern with bodily conduct, such as swallowing or handling over material objects, and show how the body acts in ways that hinge on participants' verbal conduct. The other two papers start with a concern with language, demonstrating that linguistic structures, the related actions, and their interactional consequences, cannot be fully understood if analyzed by way of extracting these structures from the very ecologies of their actual use.

Based on their American English data, Fox and Heinemann analyze the manual handing over of objects in a shore-repair shop in terms of turn-taking of the participants' hands. Results show that participants orient to “one person touches at a time” as evident in their minimizing gaps and overlaps in the handing over, and that the very object-transfer is also coordinated with verbal conduct, being typically placed after the repair-request sequence. The study hence investigates turn-taking beyond the verbal modality.

Ogden analyzes how swallowing, a complex physical process, works in conjunction with speech in social interaction. Based on data in British English, he shows how the semiotic affordances of the audible and visible aspects of swallows can be exploited for practical interactional purposes, such as displaying affective stance, projecting more talk to come or yielding a turn. Swallowing is shown to be sensitive to sequential, syntactic and prosodic structures and to the progressivity of talk. This study contributes to our understanding of the interface between physiology and speaking.

Oloff's paper investigates the use of the Czech particle *jako* (‘like’/‘as’) as a tag-like element that clusters in multi-unit turns expressing subjective stance and mobilizes affiliative responsive actions together with multimodal displays. The paper focuses on its apparent fuzzy or “filler” uses and argues for the potential of *jako* to open up “interactive turn spaces”, which can be linked to the comparative meaning of the original conjunction.

Siitonen et al. explore the Finnish second person imperative form of *kato* “look” in interaction in nature and show how it is used for noticings together with the mentioning of a new object to be seen, in showings to launch evaluative courses of action, or as prompts in which the recipients are guided to do something relevant with the target. Especially the latter heavily rely on spatial and embodied aspects rather than verbal resources, yet again showcasing the central importance of context in language use.

## Conclusion

The contributions to this Research Topic advance our knowledge of the infrastructure of human interaction, such as turn-taking, projection, and action ascription. They provide novel insights into the complex temporalities of different semiotic systems and their conjoint contribution to action formation and ascription. We hope they will stimulate future research on the grammar-body interface in social interaction.

## Author Contributions

All authors listed have made a substantial, direct, and intellectual contribution to the work and approved it for publication.

## Funding

This collection of studies was brought together with the generous support of the Swiss National Science Foundation, as part of the project the Emergent Grammar of Clause Combining in Social Interaction, grant no. 100012_178819 (SP), and the Swedish Research Council, as part of the project Vocal Practices for Coordinating Human Action, VR 2016-00827 (LK).

## Conflict of Interest

The authors declare that the research was conducted in the absence of any commercial or financial relationships that could be construed as a potential conflict of interest.

## Publisher's Note

All claims expressed in this article are solely those of the authors and do not necessarily represent those of their affiliated organizations, or those of the publisher, the editors and the reviewers. Any product that may be evaluated in this article, or claim that may be made by its manufacturer, is not guaranteed or endorsed by the publisher.

